# Diffusion tensor imaging in metachromatic leukodystrophy

**DOI:** 10.1007/s00415-018-8765-3

**Published:** 2018-01-30

**Authors:** Diane F. van Rappard, Marsh Königs, Marjan E. Steenweg, Jaap Jan Boelens, Jaap Oosterlaan, Marjo S. van der Knaap, Nicole I. Wolf, Petra J. W. Pouwels

**Affiliations:** 1 Department of Pediatric Neurology, Center for Childhood White Matter Disorders, VU University Medical Center, Amsterdam, The Netherlands; 2 Amsterdam Neuroscience, VU University Medical Center Amsterdam, Academic Medical Center, VU University Amsterdam and University of Amsterdam, Amsterdam, The Netherlands; 3 Clinical Neuropsychology Section, FGB VU University, Amsterdam, The Netherlands; 4 Emma Children’s Hospital, Academic Medical Center Amsterdam, Amsterdam, The Netherlands; 5 Department of Pediatrics, Blood and Marrow Transplantation Program, University Medical Center Utrecht, Utrecht, The Netherlands; 6 Department of Pediatrics, VU University Medical Center Amsterdam, Amsterdam, The Netherlands; 7 Department of Functional Genomics, Center for Neurogenomics and Cognitive Research, VU University, Amsterdam, The Netherlands; 8 Department of Radiology and Nuclear Medicine, VU University Medical Center, Amsterdam, The Netherlands

**Keywords:** Diffusion weighted imaging, Axial diffusivity, Radial diffusivity, Sulfatide storage, White matter disorder

## Abstract

**Objective:**

We aimed to gain more insight into the pathomechanisms of metachromatic leukodystrophy (MLD), by comparing magnitude and direction of diffusion between patients and controls at diagnosis and during follow-up.

**Methods:**

Four late-infantile, 16 juvenile and 8 adult onset MLD patients [of which 13 considered eligible for hematopoietic cell transplantation (HCT)] and 47 controls were examined using diffusion tensor imaging. Fractional anisotropy (FA), mean diffusivity (MD), axial diffusivity (AD) and radial diffusivity (RD) were quantified and compared between groups using tract-based spatial statistics (TBSS). Diffusion measures were determined for normal-appearing white matter (NAWM), corpus callosum, thalamus (all based on subject-wise segmentation), and pyramidal tracts, determined with probabilistic tractography. Measures were compared between HCT-eligible patients, non-eligible patients and controls using general linear model and nonparametric permutation analyses (randomise) for TBSS data, considering family-wise error corrected *p* < 0.05 significant.

**Results:**

Throughout white matter (WM), FA was decreased and MD and RD increased in both patient groups compared to controls, while AD was decreased in NAWM and corpus callosum. In the thalamus, no differences in FA were observed, but all diffusivities were increased in both patient groups. Differences were most pronounced between controls and patients non-eligible for HCT. Longitudinally (median follow-up 3.9 years), diffusion measures remained relatively stable for HCT-treated patients, but were progressively abnormal for non-eligible patients.

**Interpretation:**

The observed diffusion measures confirm that brain microstructure is changed in MLD, reflecting different pathological processes including loss of myelin and sulfatide accumulation. The observation of both increased and decreased AD probably reflects a balance between myelin and axonal loss vs. intracellular sulfatide storage in macrophages, depending on region and disease stage.

**Electronic supplementary material:**

The online version of this article (10.1007/s00415-018-8765-3) contains supplementary material, which is available to authorized users.

## Introduction

Metachromatic leukodystrophy (MLD, OMIM 250100) is an autosomal recessive lysosomal disorder caused by mutations in the *ARSA* gene. This results in deficiency of the enzyme arylsulfatase A (ASA), essential for sulfatide metabolism [1]. Sulfatides are major myelin lipids; their accumulation, mainly in membranes, leads to demyelination and subsequently storage in macrophages that cannot digest them [1]. MLD is a devastating disease: without treatment, eventually all acquired skills are lost and patients die.

MLD has three clinical subtypes, based on age of onset. The late-infantile form starts before 30 months, usually presenting with motor deterioration. The juvenile form presents with a combination of motor and cognitive decline before 16 years. The adult form begins with cognitive decline and psychiatric symptoms thereafter [2]. When performed early, hematopoietic cell therapy (HCT) has promising results, especially for juvenile and adult patients [3, 4].

Brain magnetic resonance imaging (MRI) in MLD is characterized by bilateral symmetric T2 signal hyperintensities, starting in the corpus callosum and subsequently involving periventricular, central and subcortical white matter (WM), in addition to projection fibers such as the corticospinal tract, and eventually cerebellar WM [5]. Thalamic volume and signal intensity on T2-weighted images in the thalamus are decreased already at diagnosis [6, 7]. Typical for MLD are stripes of low signal intensity throughout the hyperintense signal on T2-weighted images in the cerebral WM, related both to the accumulation of macrophages bursting with undigested lipids and to better preserved perivascular myelin [8].

Brain diffusion tensor imaging (DTI) is based on the motion of water molecules, which is more restricted perpendicular to than along WM fibers, a feature termed diffusion anisotropy [9]. Magnitude and direction of diffusivity are determined by molecules, membranes and microtubules, and provide information about tissue composition and microstructure and its architectural organization [10, 11].

The tensor model is a relatively simple model using diffusion-weighted images (DWI) obtained with one *b* value. It results in axial diffusivity (AD), radial diffusivity (RD) and the derived fractional anisotropy (FA) and mean diffusivity (MD) [11]. Often, increased RD is thought to be correlated with myelin degradation and changes in AD to axonal degeneration or inflammation and gliosis [12–17], but it is difficult to unequivocally associate the interpretation of diffusivity variations with specific biophysical changes [18].

The precise pathomechanisms involved in MLD, such as importance of inflammation or how accumulated sulfatides lead to demyelination, are not completely understood. DTI is, taking into account its recognized limitations, a valuable tool to gain more insight into changes in tissue properties in MLD. We, therefore, compared diffusion measures (FA and the three diffusivities) between patients who were eligible for HCT, patients not eligible at time of diagnosis, and controls. HCT-eligible patients are typically in an early disease stage, while patients not eligible for HCT have more advanced disease with extensive demyelination of the WM. We also studied the longitudinal behavior of diffusion measures of both treated and untreated patients.

## Methods

### Patients and control subjects

All 28 MLD patients (4 late-infantile, 16 juvenile, and 8 adult onset), visiting the Center for Childhood White Matter Disorders, who underwent a quantitative MRI protocol at time of diagnosis between January 2007 and April 2017 were included in this retrospective study, in addition to 47 control subjects in the same age range (Table 1), after informed consent. The study was approved by the institutional review board. Diagnosis of MLD was established by brain MRI, ASA activity and *ARSA* mutation analysis [4]. Motor function was scored by the MLD Gross Motor Function (MLD-GMF) at baseline and at latest clinical follow-up [19]. Cognitive function was evaluated through neuropsychological examination using the Wechsler Intelligence scale for Children or Adults, as appropriate for age. Eligibility for HCT was based on patients’ neurological examination (no major abnormalities and able to walk independently) and cognitive function (IQ > 75). Treatment with HCT was performed as described before [4]. Characteristics of individual patients are described in Table 2. Thirteen patients were considered eligible for HCT, and 15 non-eligible for HCT. Fourteen patients received HCT (2 patients initially classified as non-eligible; one eligible patient declined HCT) [4]. Follow-up MRI examinations were available for 17 patients (12 HCT-eligible, 5 non-eligible). Median follow-up time between first and last MRI examination was 3.9 years (range 2.5 months to 11.2 years).

**Table 1:** Demographic information

	Controls	All MLD patients	Eligible for HCT	Not eligible for HCT	Contrasts *p*
Number of subjects	47	28	13	15	ns
Male/female	23/24	9/19	6/7	3/12	
Age at first scan (mean, SD, years)	10.5 (5.3)	14.5 (9.5)	16.9 (10.6)	12.4 (8.3)	0.017^a^
Late-infantile/juvenile/adult MLD	na	4/16/8	2/5/6	2/11/2	ns

*ns* not significant, *na* not applicable

^a^Post hoc Dunnett’s T3 revealed no significant pairwise group differences

**Table 2:** Characteristics of MLD patients

Patient	MLD type	Age (years)	Baseline scan (T)	Follow-up scans (*n*)	Eligible for HCT	HCT-treated
045	Late-infantile	2.0	1.5	3^b^	Yes	Yes
050	Late-infantile	2.1	1.5	1^b^	Yes	Yes
057	Late-infantile	2.4	3	0	No	No
026	Late-infantile	2.6	1.5	0	No	No
016	Juvenile	6.5	1.5	3^d^	Yes	Yes
039	Juvenile	7.0	1.5	1^c^	No	No
029	Juvenile	7.1	1.5	0	No	No
053	Juvenile	7.1	1.5	1^b^	No	Yes
005	Juvenile	7.2	1.5	1^c^	No	No
065	Juvenile	7.4	3	2^c^	Yes	Yes
064	Juvenile	8.6	3	0	No	No
006	Juvenile	8.6	1.5	1^c^	No	No
054	Juvenile	12.5	1.5	1^c^	No	No
060	Juvenile	13.1	3	0	No	No
058	Juvenile	13.8	3	3^c^	Yes	Yes
014	Juvenile	14.1	1.5	4^d^	Yes	Yes
022	Juvenile	15.1	1.5	0	No	No
067	Juvenile	17.6	3	0	Yes	Yes
068	Juvenile	19.2	3	0	No	No
061	Juvenile	20.1	3	0	No	No
021	Adult	17.8	1.5	3^d^	Yes	Yes
051	Adult	22.5	1.5	0	No	Yes
063	Adult	23.1	3	1^c^	Yes	Yes
041	Adult	25.4	1.5	6^d^	Yes	Yes
032	Adult	26.9	1.5	2^d^	Yes	No
002	Adult	28.4	1.5	5^d^	Yes	Yes
056	Adult	32.5	1.5	0	No	No
015	Adult	35.2	1.5	4^d^	Yes	Yes

^a^Age at baseline examination

^b^Follow-up examinations at 1.5 T

^c^Follow-up examinations at 3 T

^d^Follow-up examinations at both 1.5 and 3 T

Control subjects at 1.5-T underwent MRI for reasons like mild developmental delay, headache; they had normal MRI and neurological examination. Controls at 3 T had experienced a non-neurological trauma and were included in a previous study [20].

### Acquisition

Between January 2007 and April 2013, 19 patients and 20 controls had a baseline examination at 1.5 T (Siemens Sonata, Erlangen, Germany). Between May 2013 and April 2017, 9 patients and 27 controls had a baseline examination at 3 T (GE MR750, Milwaukee, WI, USA).

Conventional imaging included sagittal 3-dimensional (3D)-T1 and axial FLAIR, using the same spatial resolution at both field strengths [20, 21]. FLAIR imaging was not performed for control subjects at 3 T. DTI was obtained with a multi-slice echo planar imaging sequence and isotropic 2.5 × 2.5 × 2.5 mm^3^ voxels. At 1.5 T, we obtained 1 b0 volume and 12 gradient directions with *b* value 750 s/mm^2^, 2 acquisitions, TR/TE 6700/81 ms [21]. At 3 T, we obtained 5 b0 volumes and 30 gradient directions with *b* value 750 s/mm^2^, 1 acquisition, TR/TE 5100/75 ms, and parallel imaging factor 2 [20].

### Analysis

Diffusion tensor imaging data were analyzed using FMRIB’s software library FSL after correction of eddy current distortion and subject motion. The diffusion tensor was fitted resulting in maps of FA, AD, RD and MD. Tract-based spatial statistics (TBSS) was used to align FA images from all subjects into a common space and to create a mean FA skeleton. The aligned FA images of all participants were projected onto this skeleton and fed into voxel-wise crossparticipant statistics using randomise (see “Statistical analysis”) [22].

Based on the regional differences found in the TBSS analyses, we further analyzed diffusion measures in the following regions of interest (ROIs): normal-appearing white matter (NAWM, corpus callosum and thalamus in all subjects, and abnormal cerebral WM in patients. In addition, we analyzed the pyramidal tracts, which were determined for each subject by tractography between motor cortex and cerebral peduncles (see below).

To determine these ROIs in DTI subject space, we first outlined abnormal WM on 2D FLAIR images of patients using clusterize, a semiautomatic segmentation algorithm involving iterative region growing followed by interactive selection of abnormal WM clusters [23]. The mask of abnormal WM was registered to the corresponding 3DT1, and filled with signal intensity resembling NAWM [24]. This 3DT1 image was then segmented with the FSL tools FAST [25] and FIRST [26] to obtain WM, gray matter (GM) and deep GM (DGM) structures, including the thalamus. DGM and abnormal WM were subtracted from the WM mask to obtain NAWM. ROIs for corpus callosum and cerebral peduncles were identified using the Johns Hopkins University (JHU) WM atlas defined in standard Montreal Neurological Institute (MNI) space [27]. The motor cortex was identified in MNI space using the Automated Anatomical Labeling (AAL) atlas [28]. ROIs in MNI space were warped into 3DT1 subject space after linear and non-linear registration using FSL tools FLIRT and FNIRT. All ROIs were subsequently registered to DTI subject space using nearest neighbor interpolation.

The ROIs of motor cortex and cerebral peduncles were used as seed and target for probabilistic tractography using the FSL tools bedpostx and probtrackx2 to obtain the left and right pyramidal tract [29]. Mean diffusion measures within the pyramidal tracts were determined by weighting the underlying FA and diffusivity maps by the probability of a voxel within the tract.

### Statistical analysis

Statistical analyses were performed for HCT-eligible and non-eligible patients at baseline and control subjects. Groups were compared on demographic variables using ANOVA and Chi-square tests, as appropriate. In the TBSS analysis, FA, MD, AD and RD were compared among the three groups with nonparametric permutation analysis (randomise), using age and scanner as covariate. We considered a family-wise error (FWE) corrected *p* < 0.05 significant.

General linear model analyses (ANOVA) including age and scanner as covariates were performed for a three-group comparison of diffusion measures at baseline within selected ROIs. In case of main group effects, we performed post hoc pairwise comparisons between groups, using Dunnett’s T3.

To evaluate longitudinal evolution of disease, we created scatter plots of ROI-based diffusion measures as function of age, and we qualitatively described changes for patients with follow-up examinations.

The pyramidal tracts primarily regulate motor function. We, therefore, determined Spearman rank correlations between diffusion measures of all 28 patients at baseline and motor function, determined with MLD-GMF, at latest clinical follow-up. *p* < 0.05 was considered significant.

## Results

### Baseline

Controls, HCT-eligible and non-eligible patients did not differ on demographic variables. Only for age, a main group effect was detected (*p* = 0.017), which was not reflected in post hoc testing (Table 1).

**Fig. 1:**
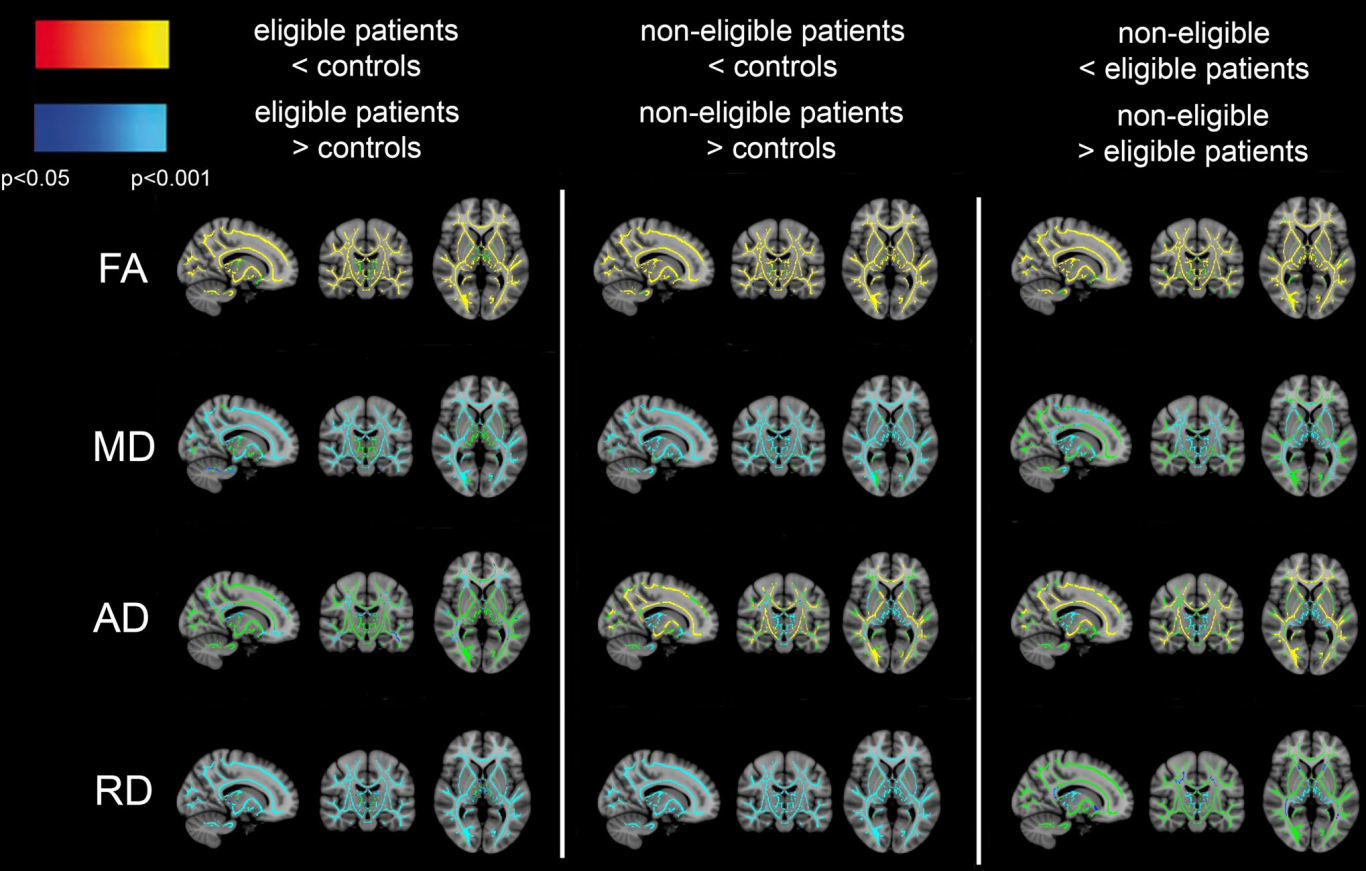
TBSS analysis for FA, MD, AD and RD comparing HCT-eligible patients vs. control subjects (left column), non-eligible patients vs. control subjects (middle column) and non-eligible vs. eligible patients (right column). FA was decreased (orange–yellow) in eligible and non-eligible patients compared to controls and in non-eligible patients compared to eligible patients in almost the whole skeleton. MD and RD were increased (blue–light blue) in both patient groups compared to controls, and in non-eligible patients compared to eligible patients. AD was increased in eligible patients compared to controls in parts of the skeleton. When comparing non-eligible patients to controls or to eligible patients, AD was increased mainly in the thalamus (blue–light blue), and decreased in WM areas including the corpus callosum (orange–yellow). The part of the WM skeleton that does not differ between groups is indicated in green. A family-wise error corrected *p* < 0.05 was considered significant. As shown in the color bar, orange and blue correspond to *p* < 0.05, while yellow and light blue correspond to *p* < 0.001

In the TBSS analysis of all baseline examinations (see Fig. 1), FA was decreased in HCT-eligible and non-eligible patients compared to controls, and in non-eligible patients compared to eligible patients in almost the entire skeleton. The differences were highly significant, since they remained present at FWE-corrected *p* < 0.001, as indicated by the yellow color. An increase of MD and RD in both patient groups compared to controls was also observed in almost the whole skeleton, again highly significant (FWE-corrected *p* < 0.001), as indicated by the light blue color. An increase of MD and RD in non-eligible patients compared to eligible patients was limited to a smaller part of the skeleton. An increase in AD in HCT-eligible patients compared to controls was restricted to part of the periventricular WM and the genu and splenium of the corpus callosum. In HCT-non-eligible patients compared to both controls and eligible patients, AD was increased mainly in the thalamus, but decreased in a large part of the skeleton, including the corpus callosum.

**Fig. 2:**
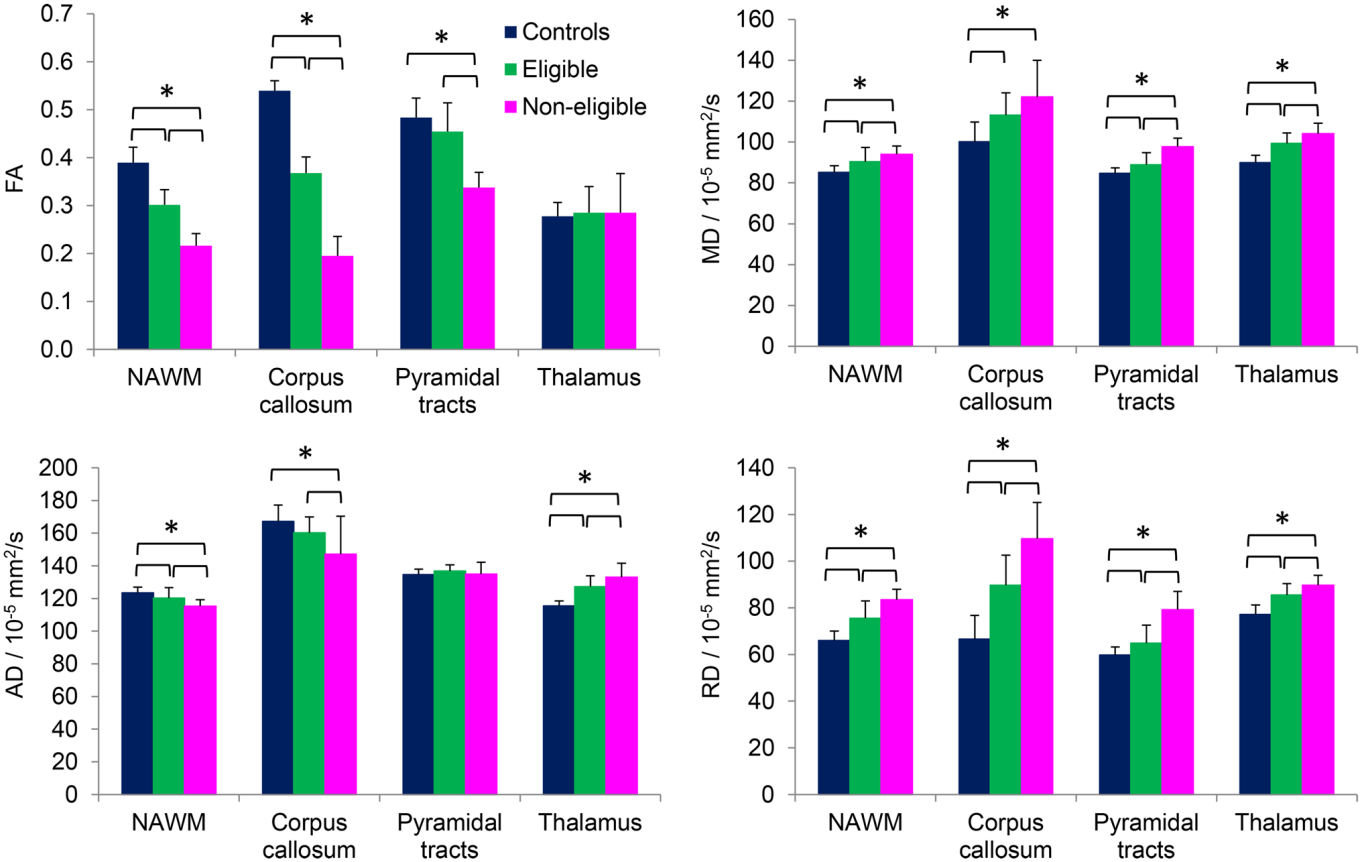
Mean values for FA, MD, AD and RD in NAWM, corpus callosum, pyramidal tracts and thalamus for control subjects (blue), eligible (green) and non-eligible (pink) patients. Error bars indicate standard deviations. Significant differences between groups are indicated with square brackets and a single asterisk (post hoc Dunnett’s T3, *p* < 0.05)

In the selected ROIs (see Fig. 2), FA was decreased for both patient groups compared to controls in NAWM, corpus callosum and pyramidal tracts. Differences were most pronounced between controls and HCT-non-eligible patients. The relative and absolute decrease in FA was largest in corpus callosum. In patients, FA in abnormal WM was smaller than in NAWM, and lower in HCT-non-eligible patients than in eligible patients (supplementary material, Table 1). Although the TBSS analysis showed group differences in FA in the skeletonized thalamus, there were no FA differences in the thalamus based on a ROI analysis. The FA observations in all ROIs were replicated when splitting the groups based on field strength (supplementary material, Fig. 1).

Following the TBSS findings, MD and RD were increased in both patient groups in NAWM, corpus callosum, pyramidal tracts and thalamus, and differences were most pronounced between controls and non-eligible patients. Differences in RD were larger than differences in MD, again with the corpus callosum showing most prominent differences between groups. MD and RD within abnormal WM were higher than in NAWM, but did not differ between patient groups (supplementary material, Table 1).

In the pyramidal tracts, there were no group differences in AD. Following the TBSS findings, in NAWM and corpus callosum, AD was lower in both patient groups than in controls, whereas in the thalamus AD was higher in patients. Again, these differences were most pronounced between HCT-non-eligible patients and controls. AD within abnormal WM was higher than in NAWM, and lower in non-eligible patients than in eligible patients (supplementary material, Table 1).

We observed similar patterns for MD, RD and AD when splitting the groups based on field strength (supplementary material, Fig. 1).

Spearman rank correlations with MLD-GMF were significant for FA (− 0.84), MD (0.78) and RD (0.86), all *p* < 0.01. Thus, low FA and high MD and RD of the pyramidal tracts at baseline indicate poor motor function at follow-up.

### Longitudinal evolvement of diffusion measures

**Fig. 3:**
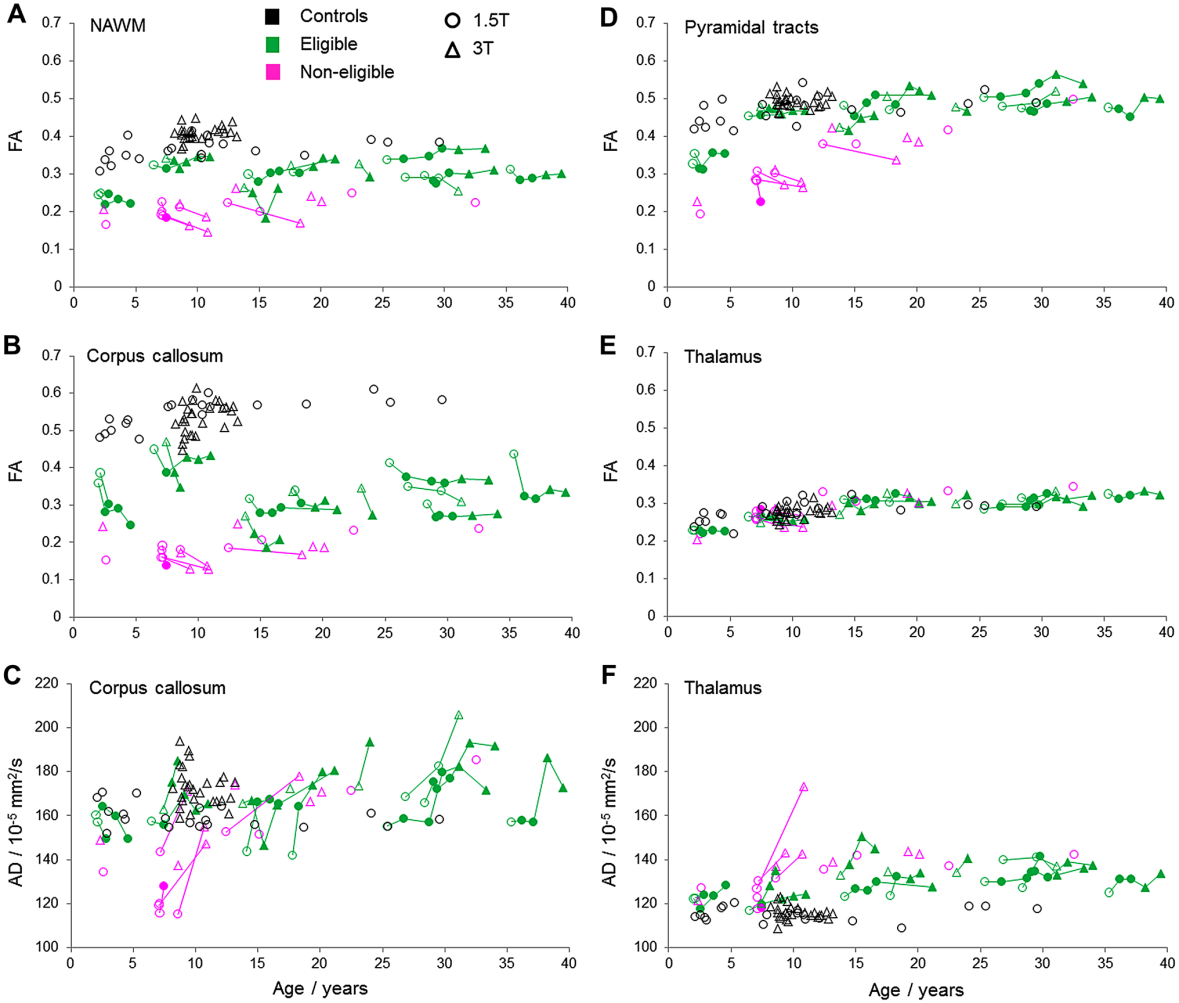
Longitudinal evolvement of **a** FA in NAWM, **b** FA and **c** AD in corpus callosum, **d** FA in pyramidal tracts, **e** FA and **f** AD in thalamus. Control values are indicated in black, HCT-eligible patients in green and non-eligible patients in pink. Data measured at 1.5 T are indicated with a circle, data measured at 3 T with a triangle. After transplantation, symbols are filled

For illustration, a selection of longitudinal diffusion measures in selected ROIs is shown in Fig. 3. For each patient with follow-up measurements, symbols are connected by lines. The longitudinal variation indicates the actual course, but also the reproducibility of the measurement, including the effect of examinations at both field strengths for some patients. The effect of field strength can also be appreciated when comparing control subjects at 3 and 1.5 T. Overall, measures remained relatively stable, especially for HCT-eligible patients after treatment. In non-eligible patients, values showed a progressively abnormal trend.

In NAWM, FA mildly fluctuated for treated eligible patients, while FA further decreased for HCT-non-eligible patients (Fig. 3a). Diffusivities remained relatively stable for all patients. In the corpus callosum, FA tended to decrease in the treated eligible patients, while the reduction in the non-eligible patients was marginal (Fig. 3b). However, MD, AD and RD longitudinally increased especially in non-eligible patients, which meant that AD, which was decreased at baseline, showed a pseudo-normalization (Fig. 3c).

In the pyramidal tracts, FA remained constant or slightly increased over time in most treated eligible patients, whereas FA slightly decreased in HCT-non-eligible patients (Fig. 3d). Diffusivities showed some longitudinal variability, but no clear trend was observed.

In the thalamus, in which FA did not differ between groups at baseline, FA remained stable in treated eligible patients, and showed a slight decrease in non-eligible patients (Fig. 3e). Diffusivities in treated eligible patients remained stable or showed a mild increase, whereas a larger increase was observed in non-eligible patients (as shown for AD in Fig. 3f).

## Discussion

Using diffusion-weighted MRI, we compared magnitude and direction of diffusion between MLD patients and controls to gain insight into the microstructure of affected brain tissue. At baseline, FA was decreased and MD and RD were increased in MLD patients compared to controls throughout the WM, not only in the corpus callosum (affected early in the disease), but also in NAWM. FA measures of the thalamus did not differ between groups, but its components AD and RD were both increased in patients compared to controls. Whereas AD was increased in thalamus, it was unchanged in the pyramidal tracts and decreased in the corpus callosum and, to a lesser degree, in NAWM. All differences were most pronounced between controls and HCT-non-eligible patients.

Longitudinally, in treated HCT-eligible patients, diffusion measures remained stable or showed only minor changes. FA remained constant or even tended to increase in NAWM, pyramidal tracts, and thalamus, whereas it slightly decreased in the corpus callosum. HCT-non-eligible patients had less follow-up examinations than eligible patients, but those available showed clear increases of RD and AD, causing a small FA reduction in all investigated regions. The treatment effect of HCT most likely influenced the longitudinal differences between treated eligible patients (approaching control values in NAWM and pyramidal tracts), and untreated non-eligible patients (increasingly abnormal values). This is in line with our observation that metabolite concentrations observed with magnetic resonance spectroscopy partially normalized in successfully transplanted patients, whilst concentrations for non-treated patients further deteriorated [30].

The diffusion tensor model used in this study reflects the underlying structural characteristics in a simplified manner, hampered by partial volume effects and crossing fibers [18]. Advanced multi-compartment diffusion models, such as the composite hindered and restricted model of diffusion (CHARMED), are more sensitive than the conventional ones [31–33]. However, since our study, ongoing since 2007, concerns a rare disease, application of these advanced diffusion models was not feasible.

This implies that we can merely hypothesize about the precise mechanism responsible for the observed differences rather than draw general conclusions because different cellular processes may lead to identical changes [9–11]. Since both animal [13, 34, 35] and human studies [36] have shown an increased RD parallel to myelin loss, our results of increased RD in WM suggest myelin loss in patients, in line with histopathological findings [37]. This is also supported by our observation that high RD and low FA in the pyramidal tracts at baseline indicate poor motor function at follow-up.

With regard to AD, animal and human studies provide discrepant results, correlating axonal damage with either an AD decrease [13, 17] or increase, [38, 39], respectively. This reflects the difficulty in relating AD to underlying pathological processes. Our observation of opposing AD patterns in MLD suggests that different pathological mechanisms can cause either a decrease or an increase in AD, with the overall balance between these effects depending on brain region and disease stage. MLD is characterized by accumulation of sulfatides, major myelin lipids mainly synthesized by oligodendrocytes. Analytical studies of MLD patients’ brain tissue showed that metachromatic deposits are mainly present in the WM, with a sulfatide content up to eight times higher than normal, with relatively minor chemical GM changes [37]. Regarding WM, we assume that the massive intracellular sulfatide accumulation in swollen macrophages, in vain trying to digest these lipids, causes overall diffusion restriction and thereby AD reduction. Using conventional DWI, restricted diffusion in the outermost part of the demyelinated WM has indeed been described for single cases in relatively early disease stage [40, 41]. However, as the disease progresses, axons are increasingly damaged. Based on the previous observations in human studies, we expect that loss of both myelin and axons will lead to an AD increase [38, 39]. Our results suggest that, particularly in the corpus callosum of non-eligible patients at baseline, diffusion restriction due to sulfatide accumulation in macrophages contributes more to the severely reduced AD values than increased diffusion due to myelin and axonal loss. Our longitudinal observation of an increase, and thereby a pseudo-normalization of AD, in the corpus callosum suggests that myelin and axonal loss likely becomes more prominent in progressive disease. The corpus callosum is, of the investigated ROIs, least hindered by limitations of the tensor model, suggesting that the interpretation of AD values is not much influenced by the presence of crossing fibers. Whether inflammation and gliosis also contribute to AD changes in this setting is uncertain.

The thalamus is a DGM structure, in which sulfatide accumulation is much more limited than in WM [37]. In addition, the thalamus has a different microstructure than WM as it largely consists of neurons and contains few axons. In control subjects, this is mirrored by low thalamic FA, as the difference between AD and RD is much smaller than for a WM structure like the corpus callosum. In the thalamus of patients, we observed an increase of AD and RD of similar relative magnitude, which had hardly any effect on FA. This increase of both AD and RD probably implies an increase in extracellular space due to neuronal loss, which apparently dominates reductions in diffusivity due to storage material.

Limitations of this study were its retrospective character, a large age range of patients (inherent to the inclusion of patients with all disease types), and a limited age range of controls at 3 T. The combination of 1.5 and 3 T data also introduced some variability, although these differences were typically smaller than differences between controls and patients. In fact, the ROI-based analysis of baseline data at 1.5 and at 3 T separately (as shown in the supplementary material) showed identical patterns as those observed in the combined analysis. We noticed the same main group effects, although in these smaller groups fewer post hoc pairwise comparisons were significant.

Overall, the observed changes of FA, RD, and especially AD indicate that MLD alters certain aspects of brain microstructure. These changes most likely reflect a multitude of pathological processes such as accumulation of metachromatic material, followed by myelin and axonal loss. The differences between untreated and treated patients indicate that diffusion measures are positively affected by HCT, further emphasizing the beneficial effects of this intervention on WM and supporting the findings of other quantitative MR measures as proton MR spectroscopy [30]. Altogether, quantitative MR measures provide more insight into time-dependent disease mechanisms and might in the future aid in determining the right window for intervention.

## Electronic supplementary material

Below is the link to the electronic supplementary material.

Supplementary material 1 (DOCX 142 kb)
