# Supplementary material for: Diffusion tensor imaging in metachromatic leukodystrophy
Source: J Neurol. 2018 Jan 30;265(3):659–68. doi: 10.1007/s00415-018-8765-3 (PMC5834549; doi:10.1007/s00415-018-8765-3)

**Supplementary data**

**Supplementary Table 1:**

Diffusion measures (mean and standard deviations) in NAWM and abnormal WM

| **Diffusion measure** | **Eligible** | | **Non-eligible** | |
| --- | --- | --- | --- | --- |
|  |  |  |  |  |
|  | NAWM | abnormal WM | NAWM | abnormal WM |
| **FA** | 0.31 (0.03) | 0.25 (0.07) | 0.22 (0.02) | 0.16 (0.03) |
| **MD** / 10^-5^ mm^2^/s | 90.4 (6.9) | 117.0 (11.5) | 94.0 (4.1) | 116.2 (11.2) |
| **AD** / 10^-5^ mm^2^/s | 120.2 (6.5) | 148.6 (11.8) | 115.3 (4.0) | 136.3 (15.6) |
| **RD** / 10^-5^ mm^2^/s | 75.5 (7.4) | 101.2 (13.8) | 83.4 (4.5) | 106.2 (9.3) |

**Supplementary Figure 1:**

Mean values for FA, MD, AD and RD in NAWM, corpus callosum, pyramidal tracts and thalamus for control subjects (blue), eligible (green) and non-eligible (pink) patients. Error bars indicate standard deviations. Significant differences between groups are indicated with square brackets and a single asterisk (post-hoc Dunnett’s T3, p<0.05).

A: Field strength 1.5T: 20 control subjects, 9 eligible patients, 10 non-eligible patients.

B: Field strength 3T: 27 control subjects, 4 eligible patients, 5 non-eligible patients.


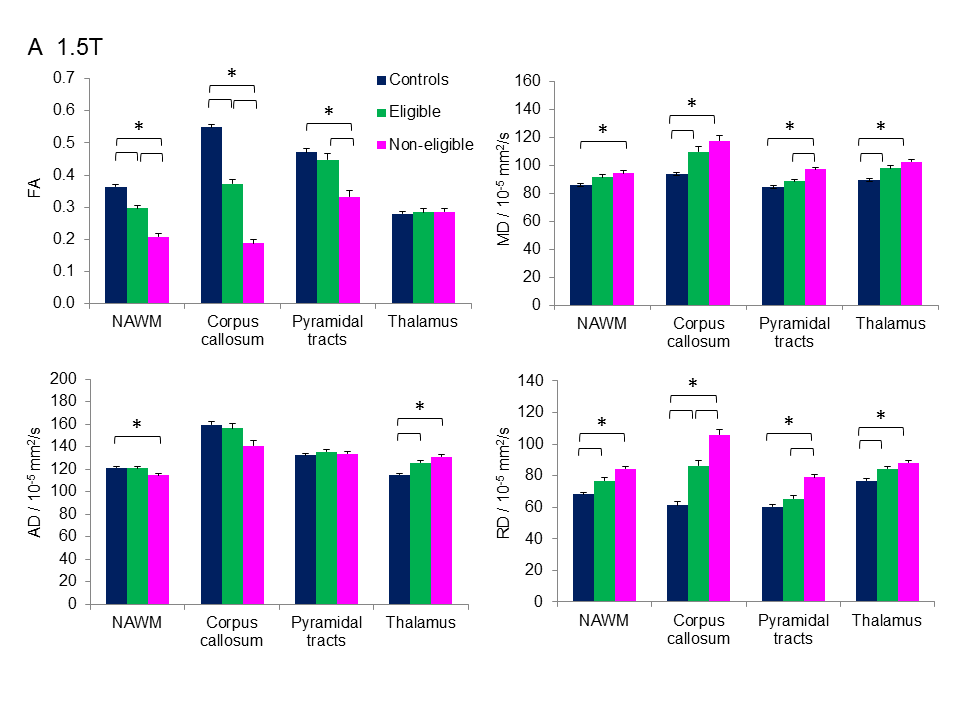

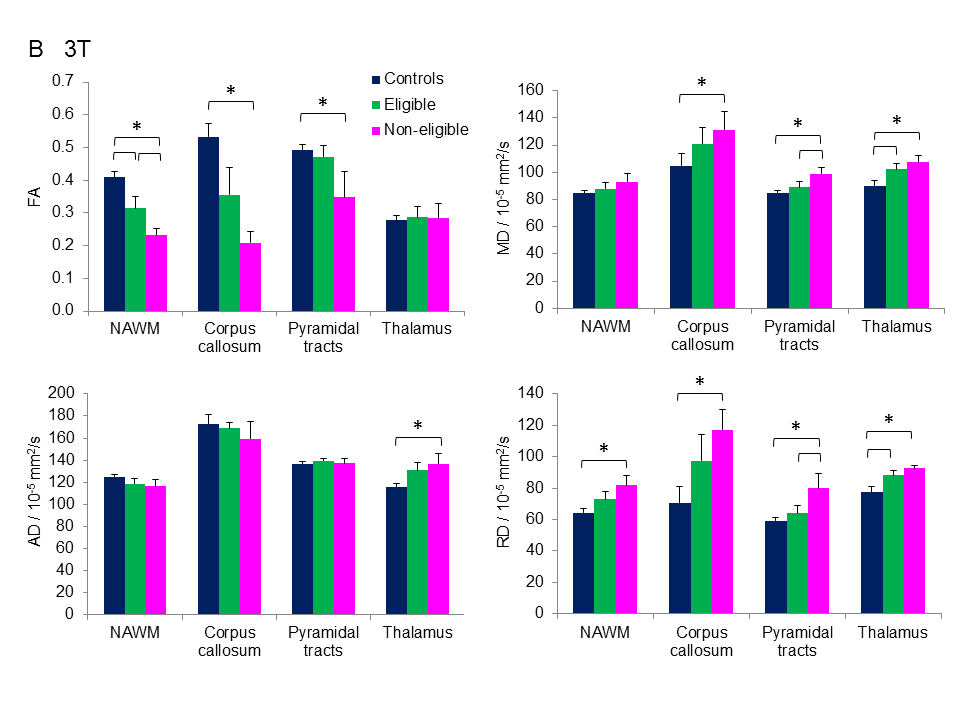

Supplement: Supplementary file 1 — Supplementary material 1 (DOCX 142 kb) [file 415_2018_8765_MOESM1_ESM.docx]
